# Correction: Study on the pathogenesis of *PLXNB1* gene in olfactory dysfunction of allergic rhinitis

**DOI:** 10.1371/journal.pone.0354708

**Published:** 2026-07-24

**Authors:** Xinglong Chen, Lingqiong Zhao, Wenlong Luo

There are errors in the Author Contributions. The correct contributions are:

**Data curation:** Xinglong Chen, Lingqiong Zhao.

**Formal analysis:** Xinglong Chen.

**Supervision:** Wenlong Luo.

**Validation:** Xinglong Chen, Lingqiong Zhao.

**Writing – original draft:** Xinglong Chen.

**Writing – review & editing:** Wenlong Luo.
