# Supplementary material for: Study on the pathogenesis of PLXNB1 gene in olfactory dysfunction of allergic rhinitis
Source: PLoS One. 2026 Jun 1;21(6):e0350102. doi: 10.1371/journal.pone.0350102 (PMC13225367; doi:10.1371/journal.pone.0350102)
Supplement: S1 — (PDF) [file pone.0350102.s001.pdf]

Control AR

GAPDH

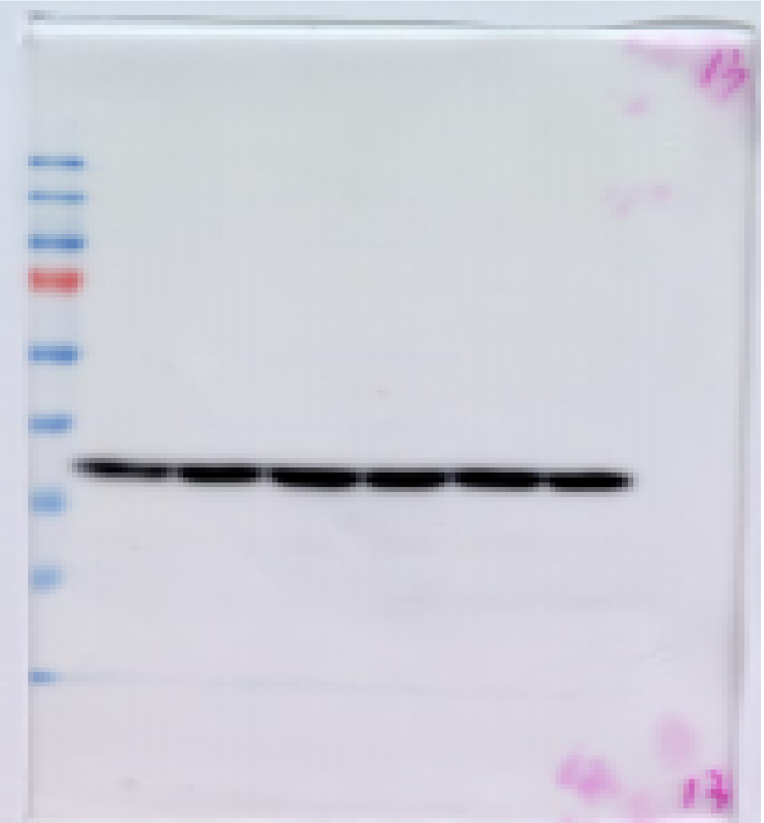

Control AR

PLXBN1

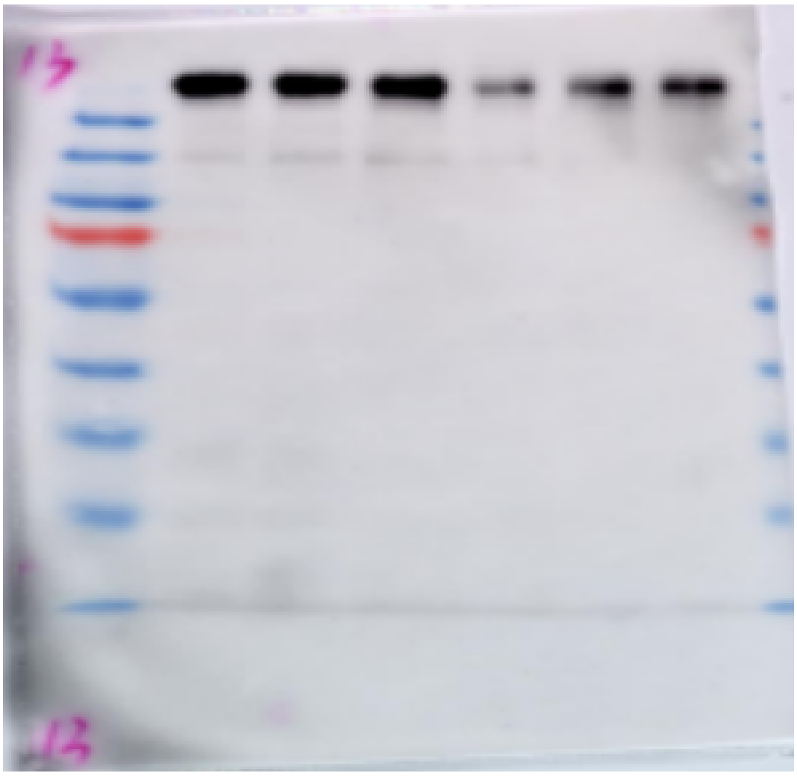

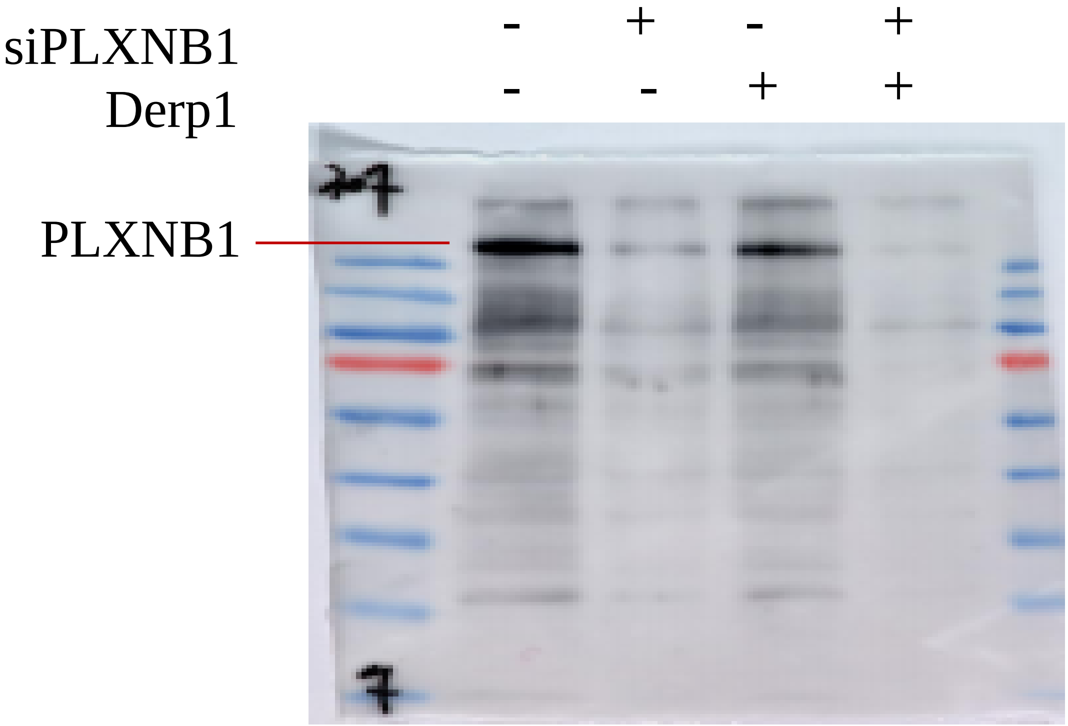
siPLXNB1 - + - +
Derp1 - - + +
PLXNB1

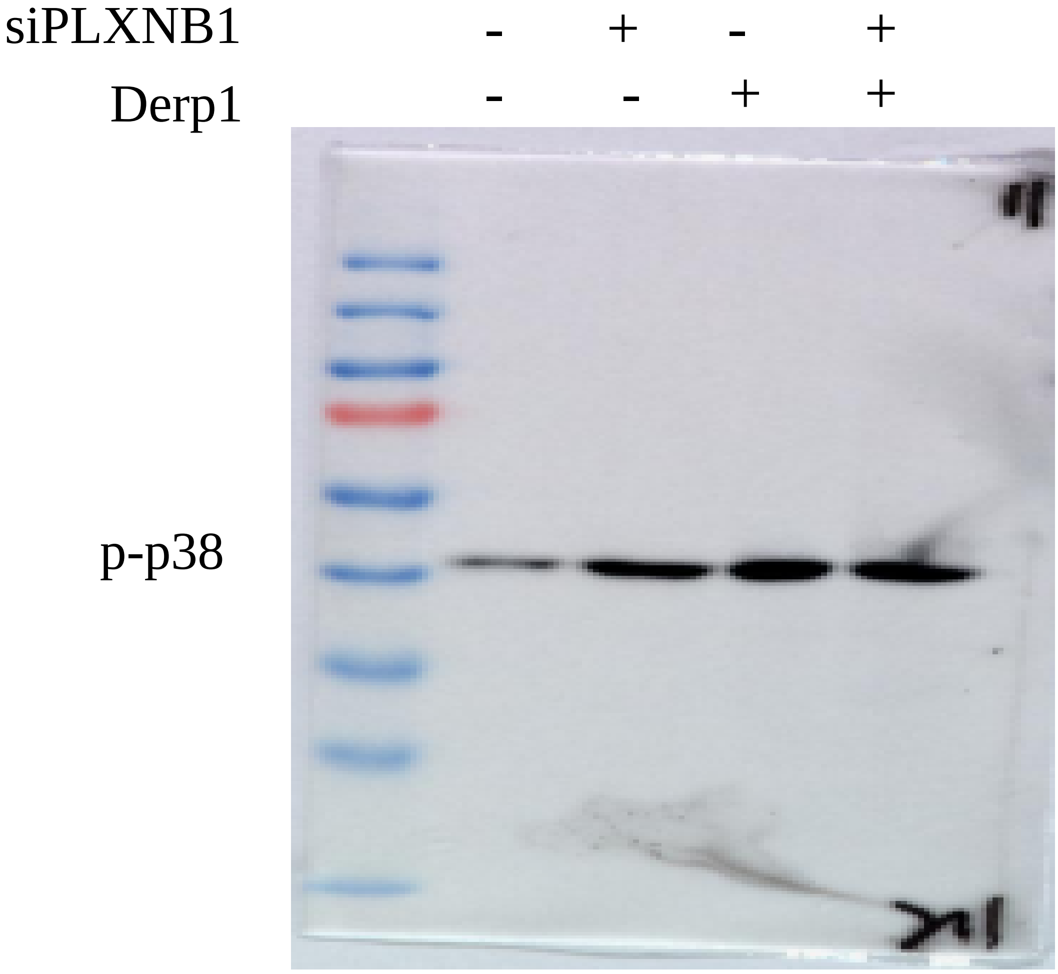
siPLXNB1 - + - +
Derp1 - - + +
p-p38

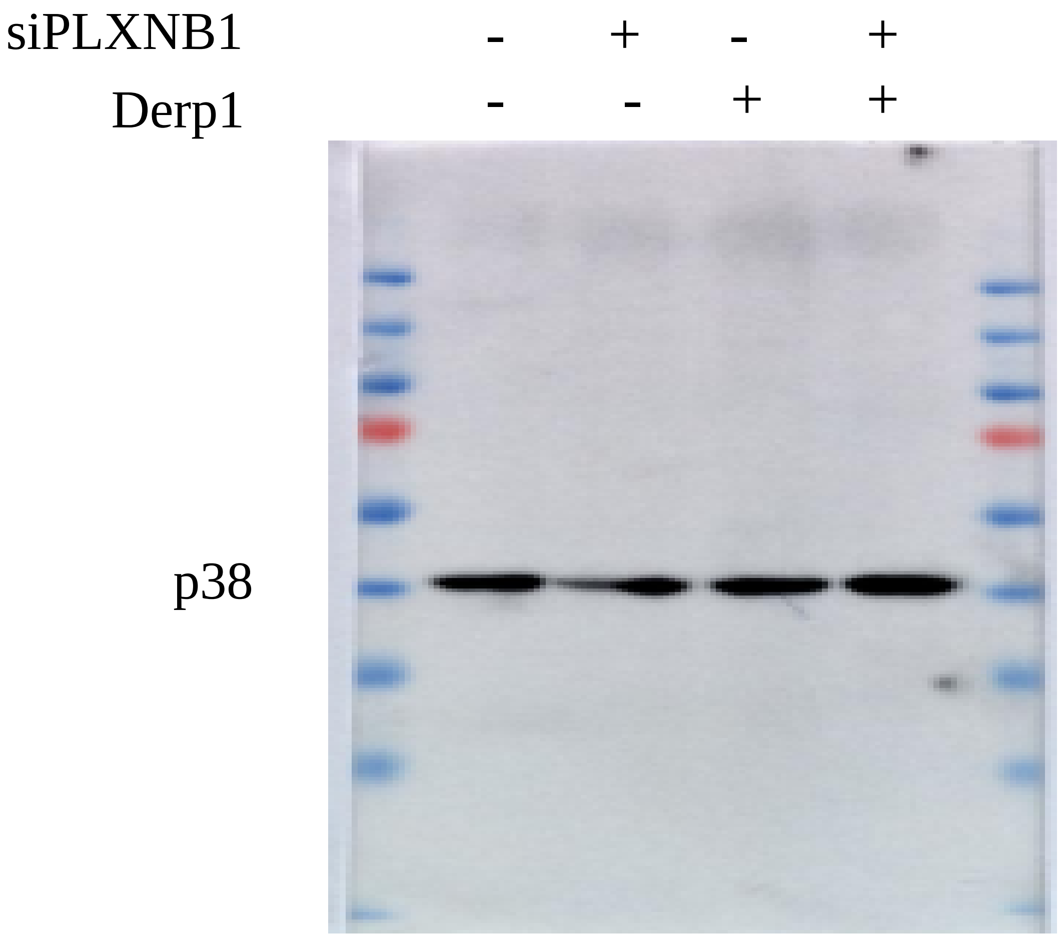
siPLXNB1 - + - +
Derp1 - - + +
p38

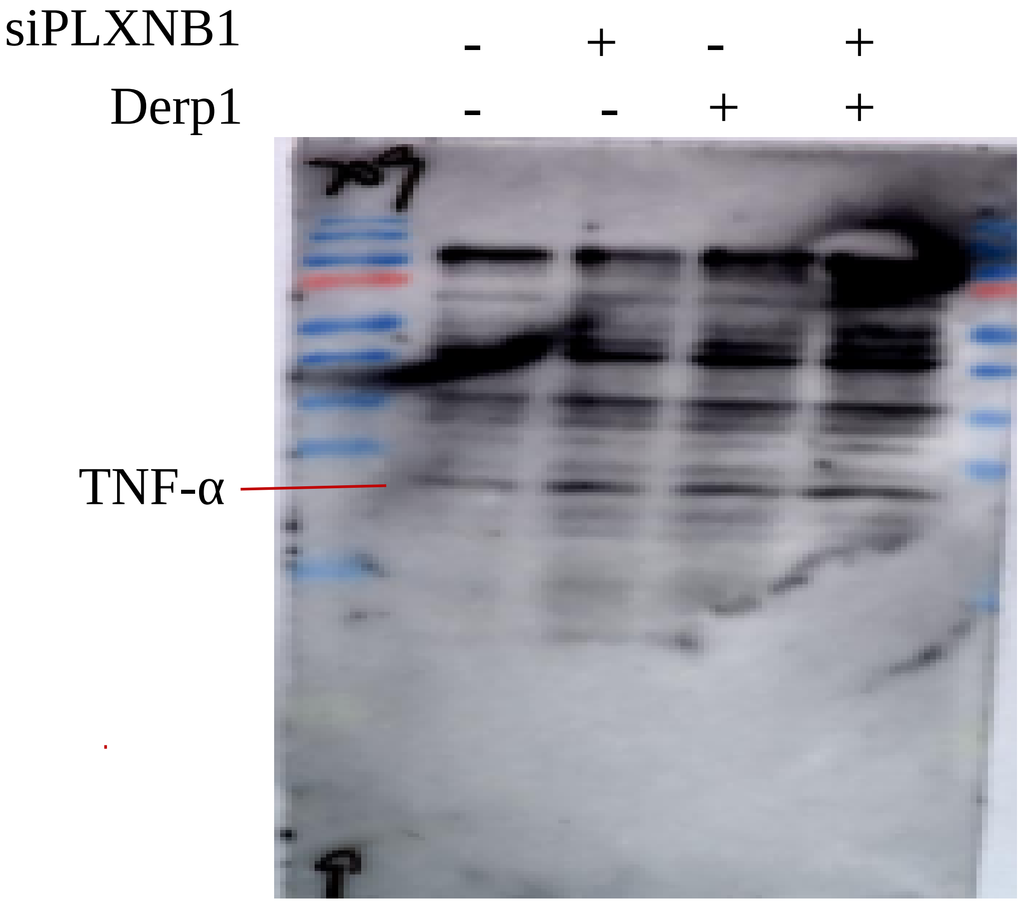
siPLXNB1 - + - +
Derp1 - - + +
TNF-α

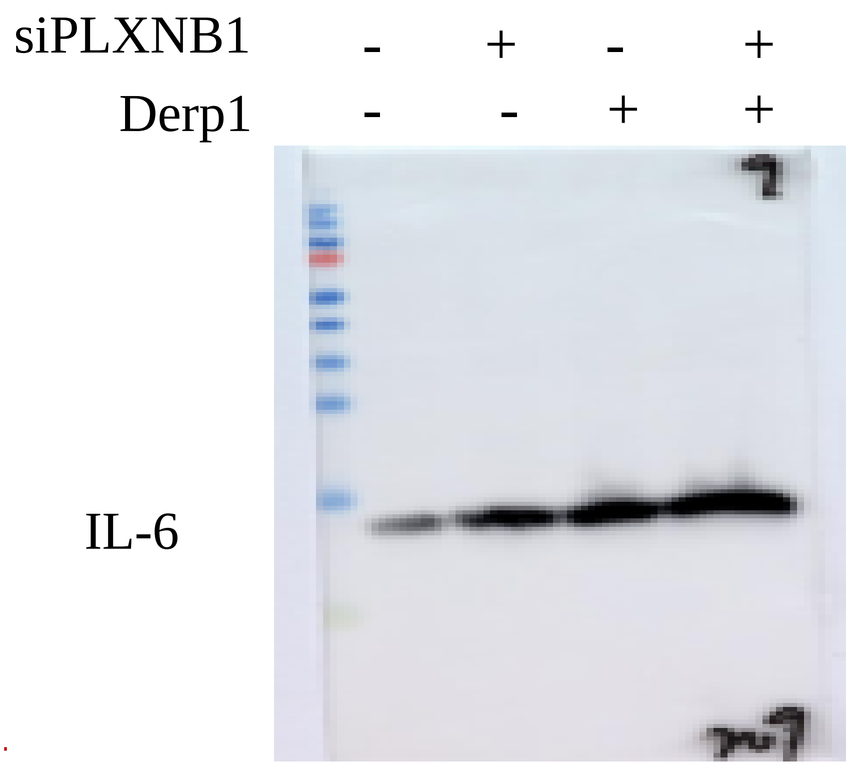
siPLXNB1 - + - +
Derp1 - - + +
IL-6

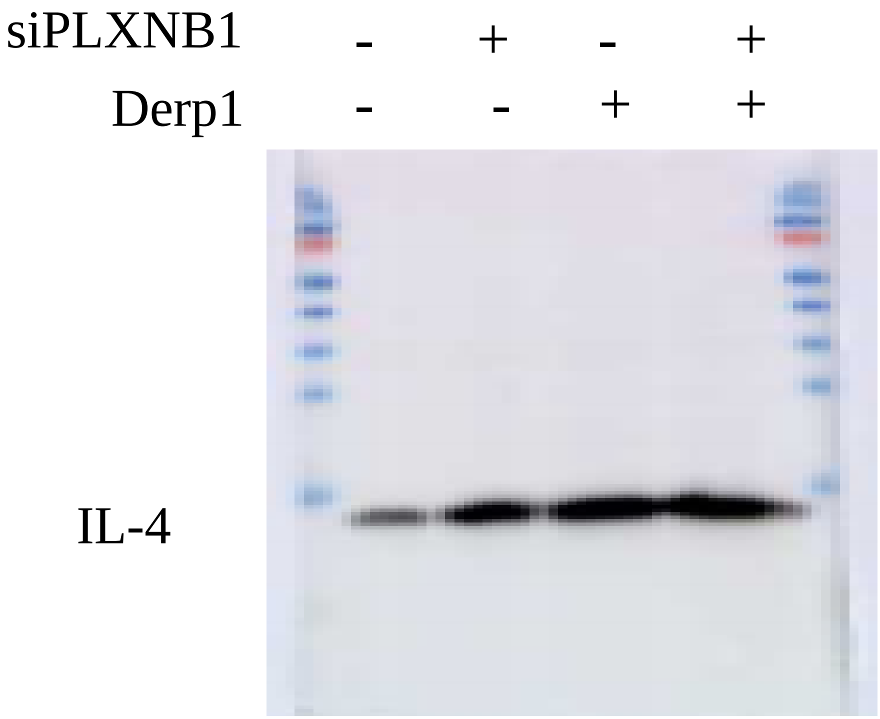
siPLXNB1 - + - +
Derp1 - - + +
IL-4

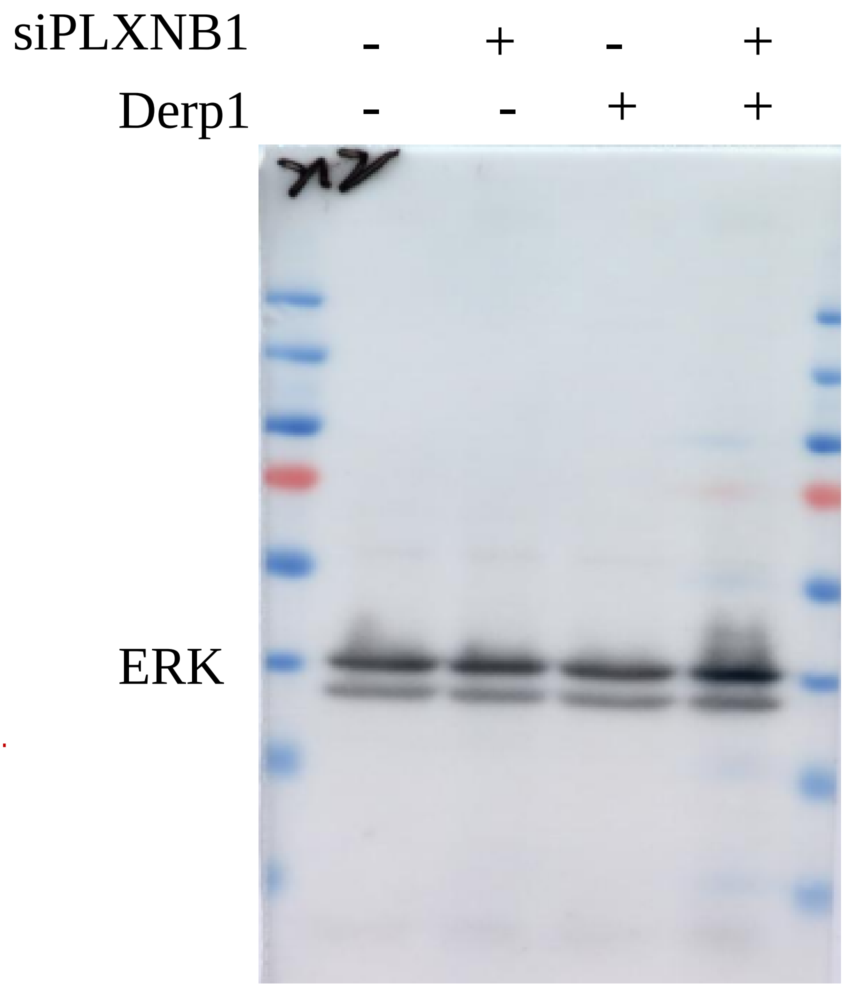
siPLXNB1 - + - +
Derp1 - - + +
ERK

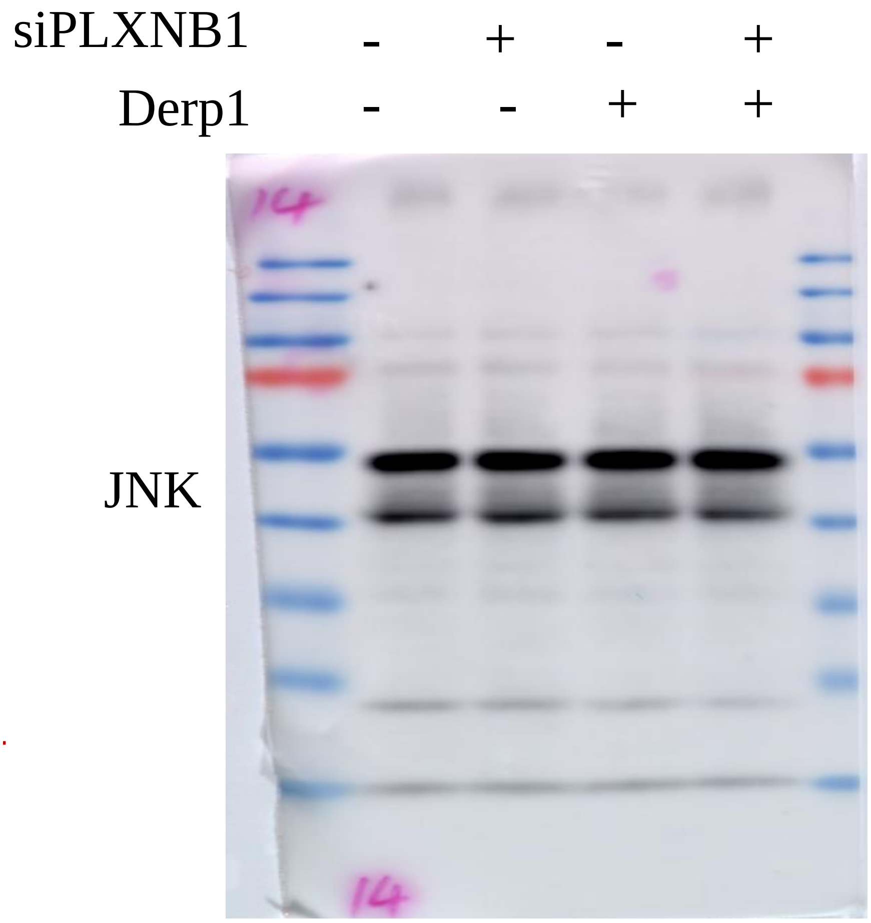
siPLXNB1 - + - +
Derp1 - - + +
JNK

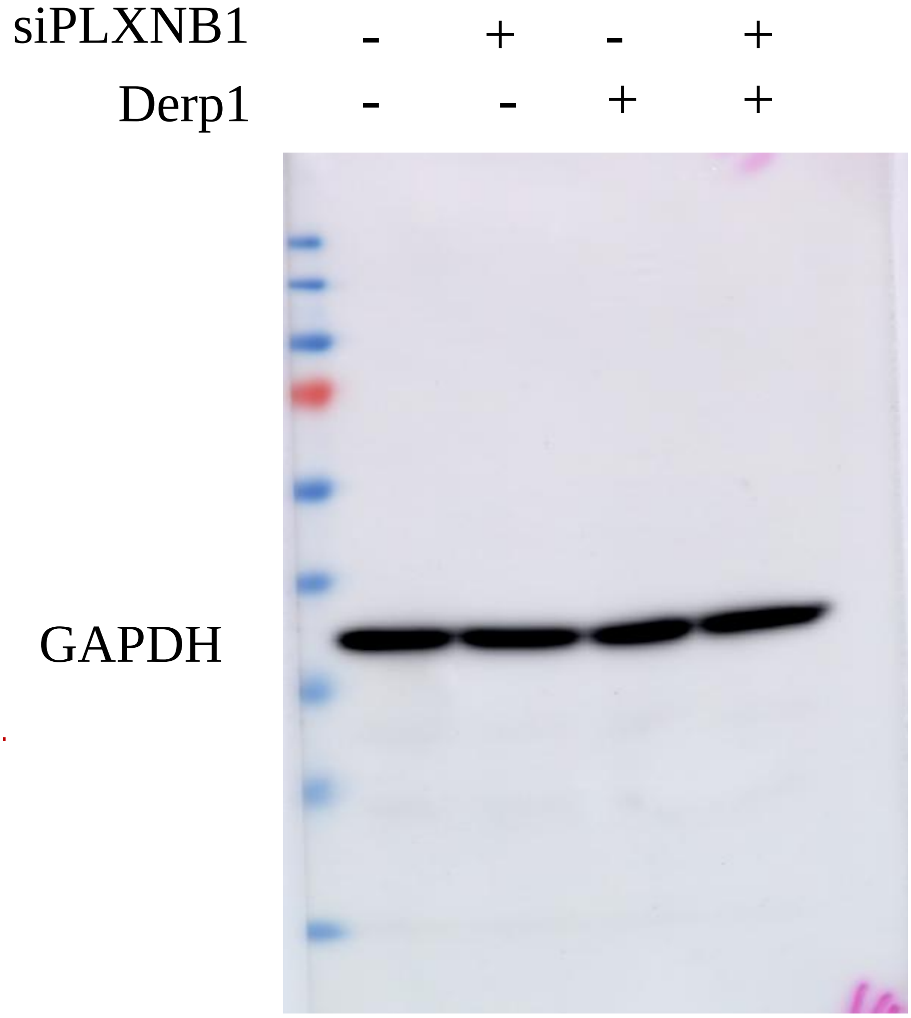
siPLXNB1 - + - +
Derp1 - - + +
GAPDH

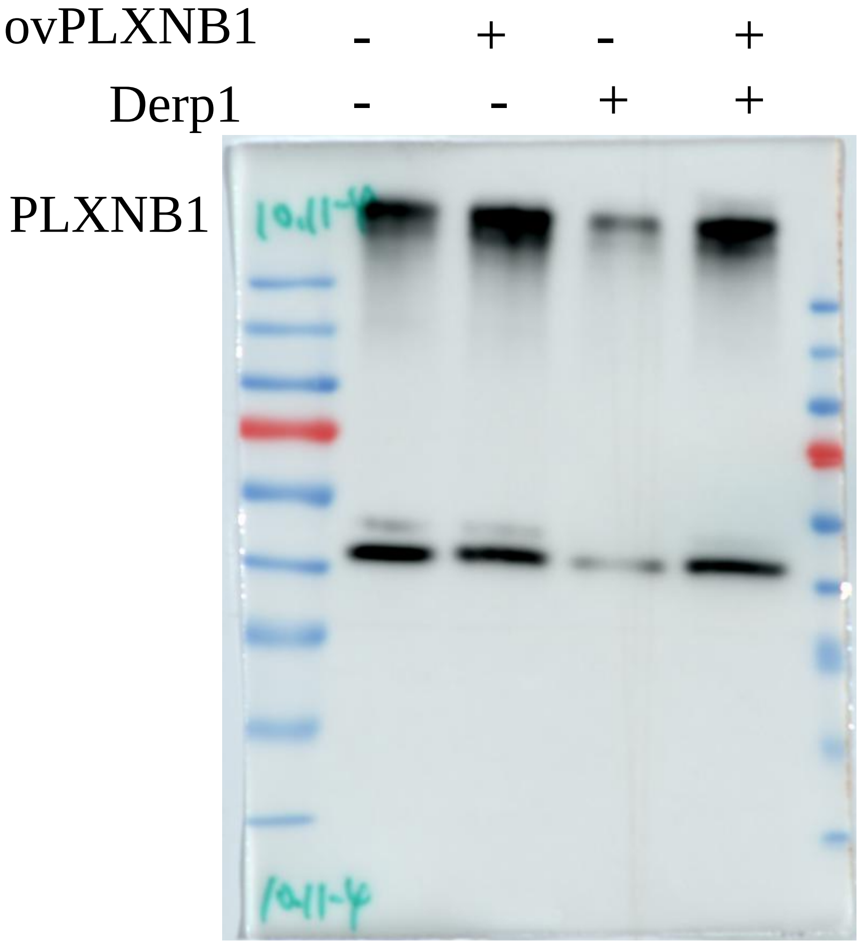
ovPLXNB1 - + - +
Derp1 - - + +
PLXNB1

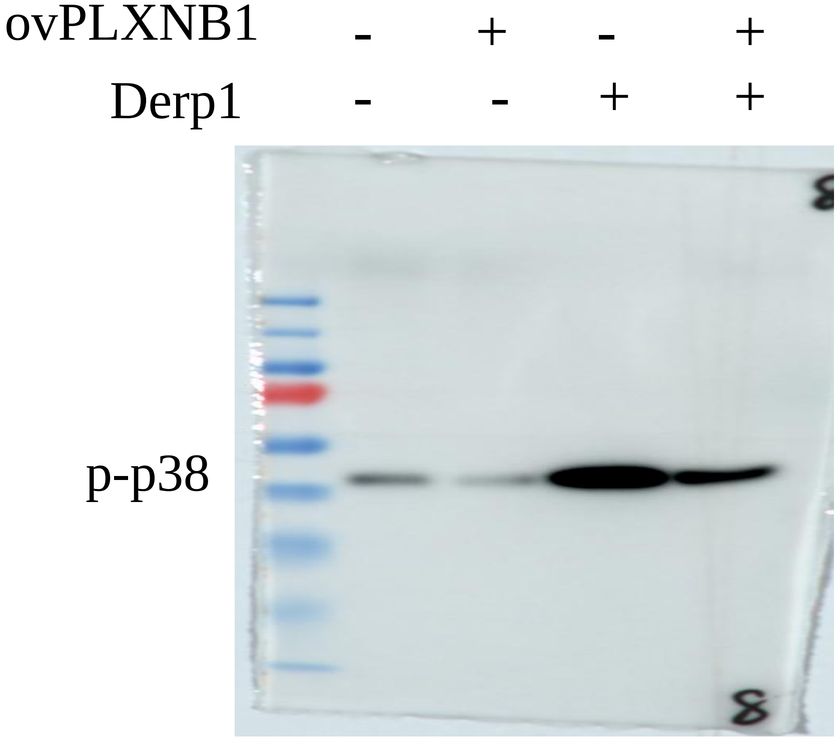
ovPLXNB1 - + - +
Derp1 - - + +
p-p38

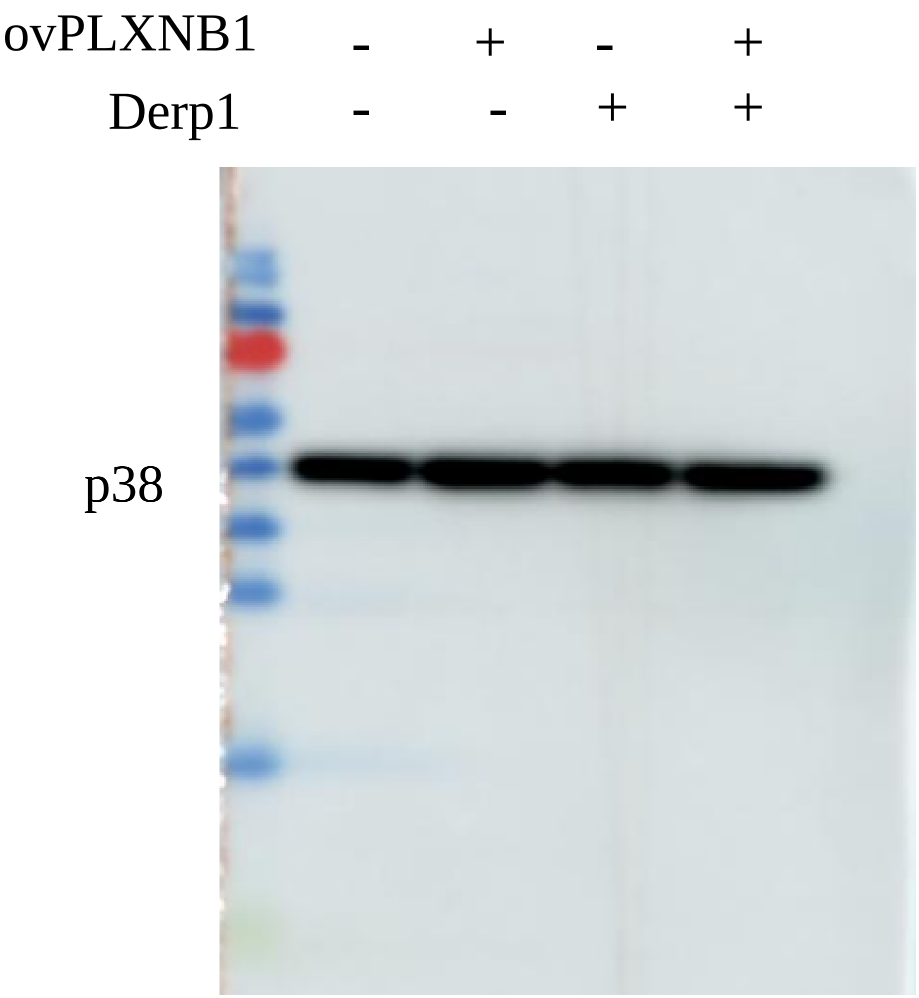
ovPLXNB1 - + - +
Derp1 - - + +
p38

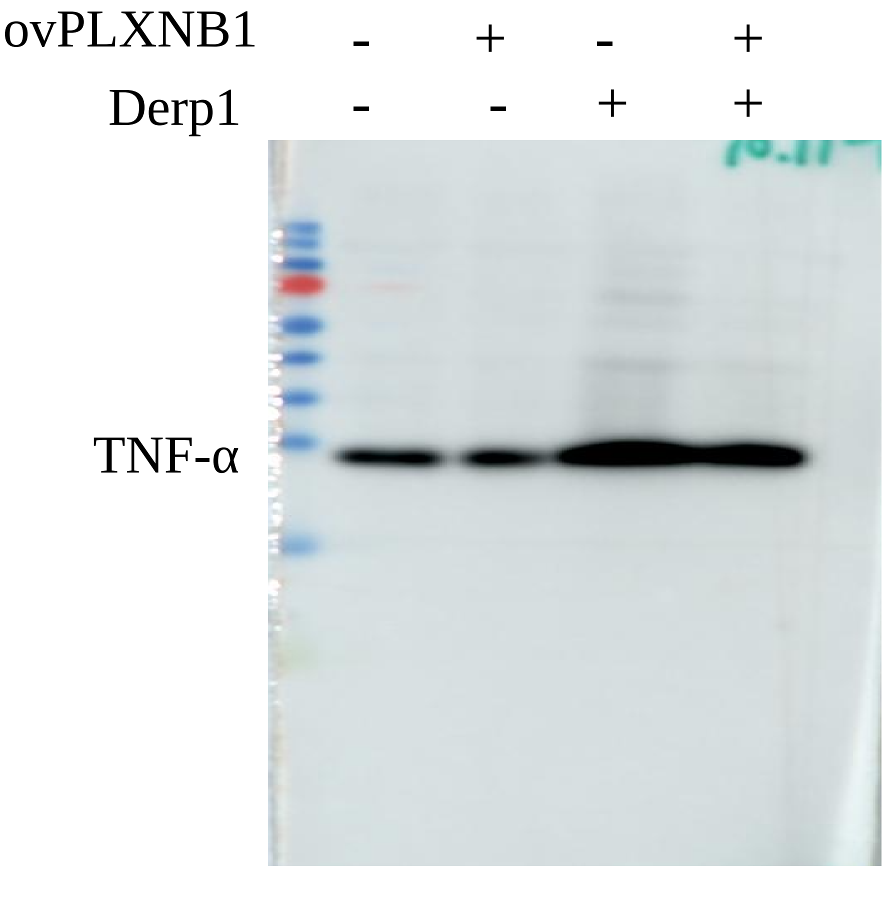
ovPLXNB1 - + - +
Derp1 - - + +
TNF-α

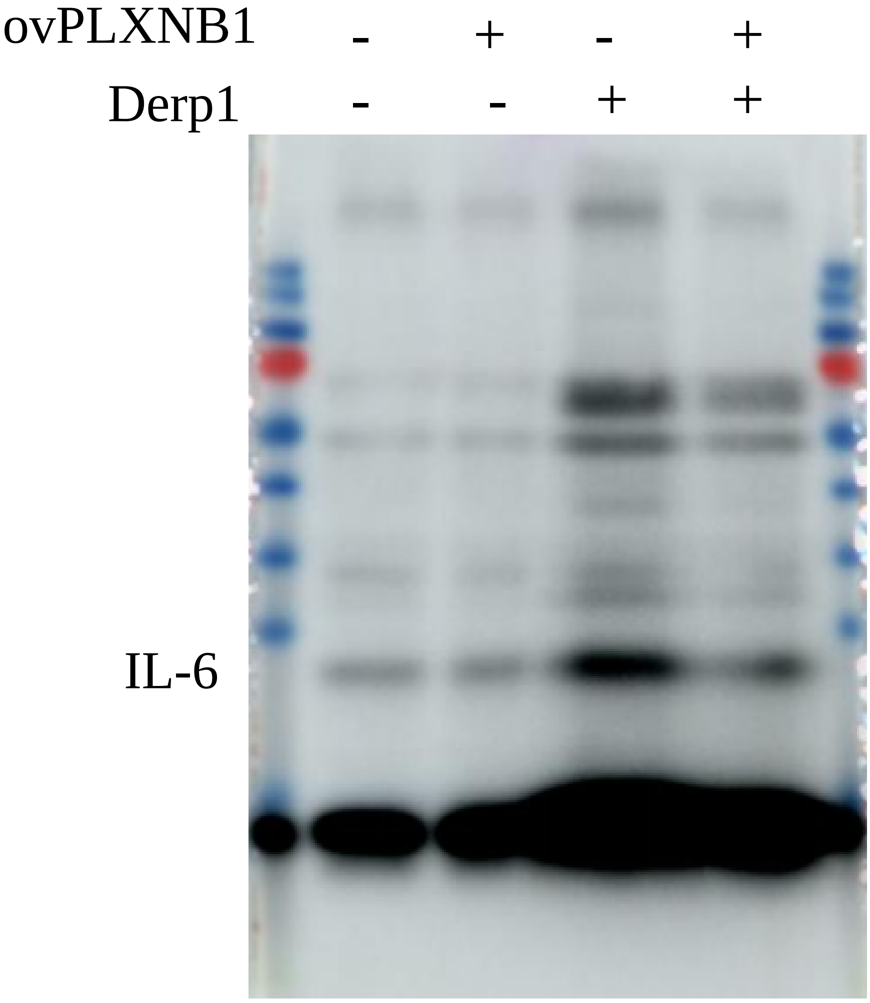
ovPLXNB1 - + - +
Derp1 - - + +
IL-6

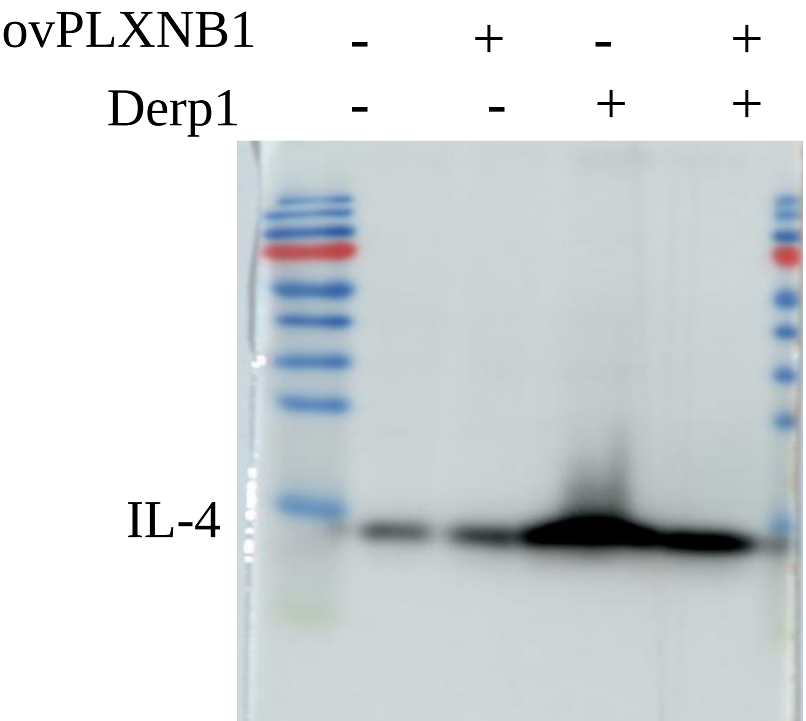
ovPLXNB1 - + - +
Derp1 - - + +
IL-4

ovPLXNB1	-	+	-	+
Derp1	-	-	+	+

ERK

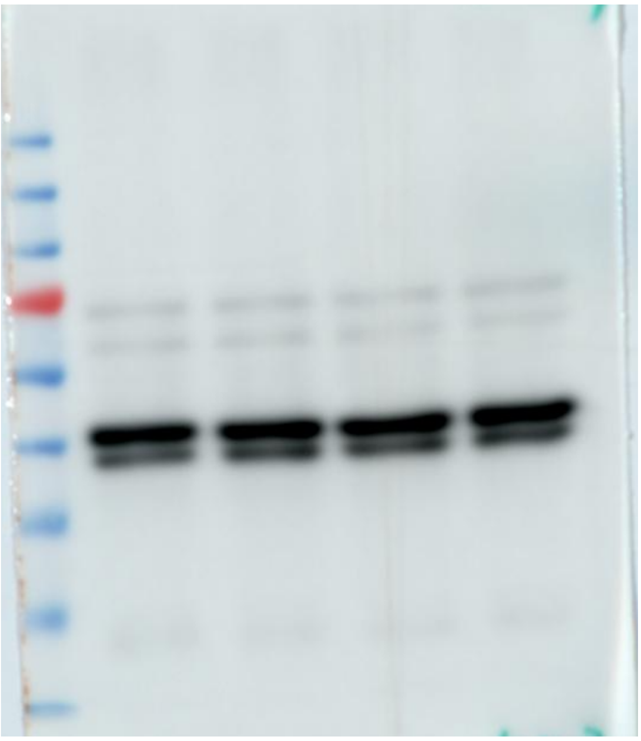

ovPLXNB1	-	+	-	+
Derp1	-	-	+	+

JNK

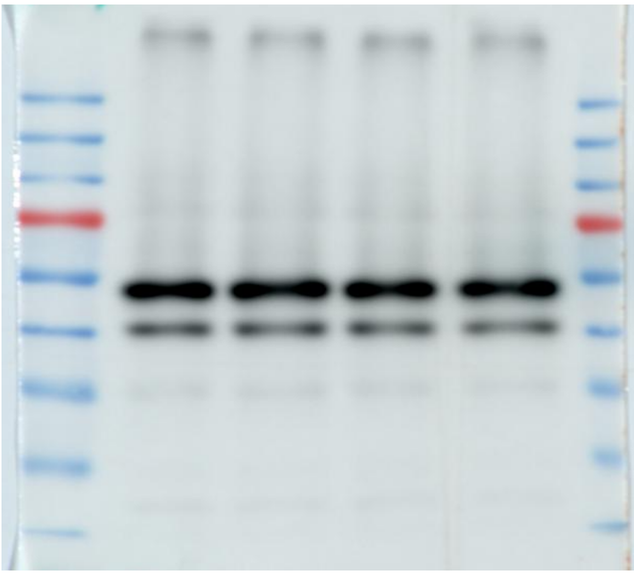

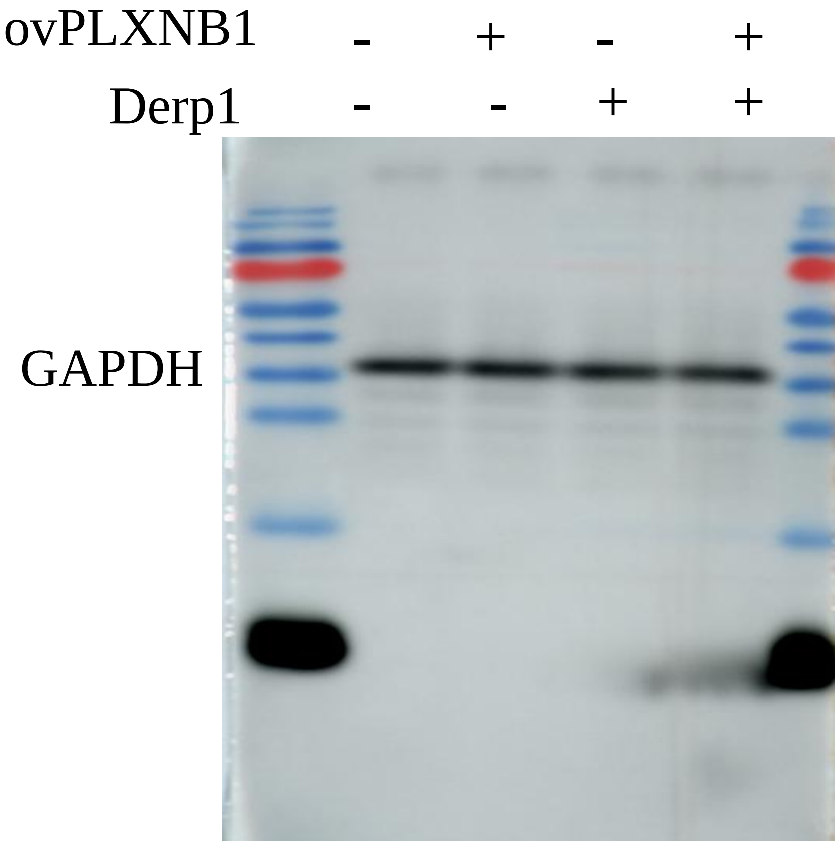
ovPLXNB1 - + - +
Derp1 - - + +
GAPDH

Desloraladine - - +
Derp1 - + +

PLXNB1

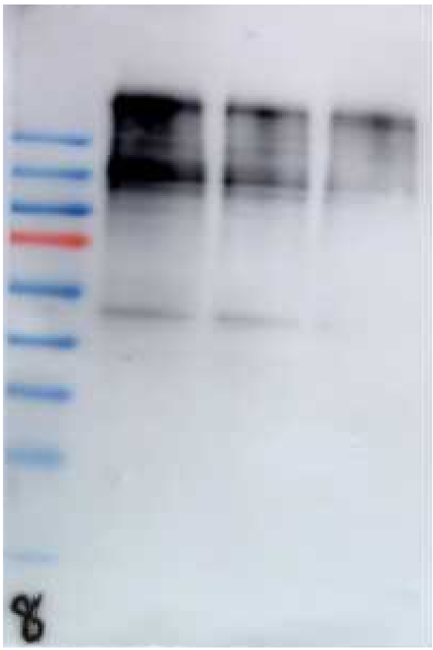

Desloraladine - - +
Derp1 - + +

TNF-α

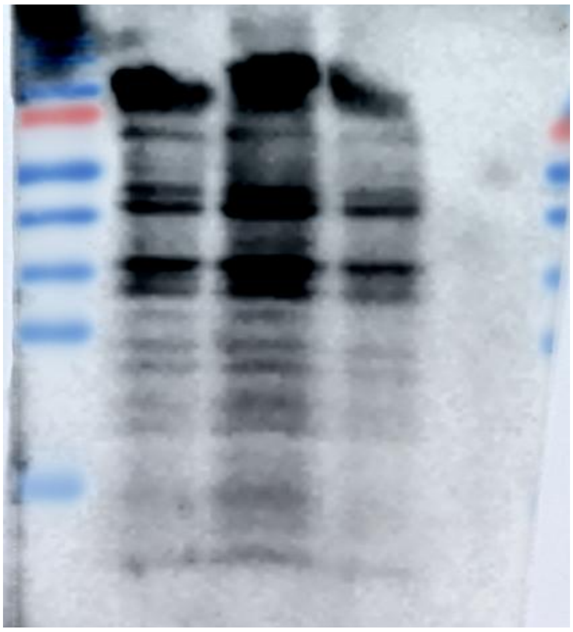

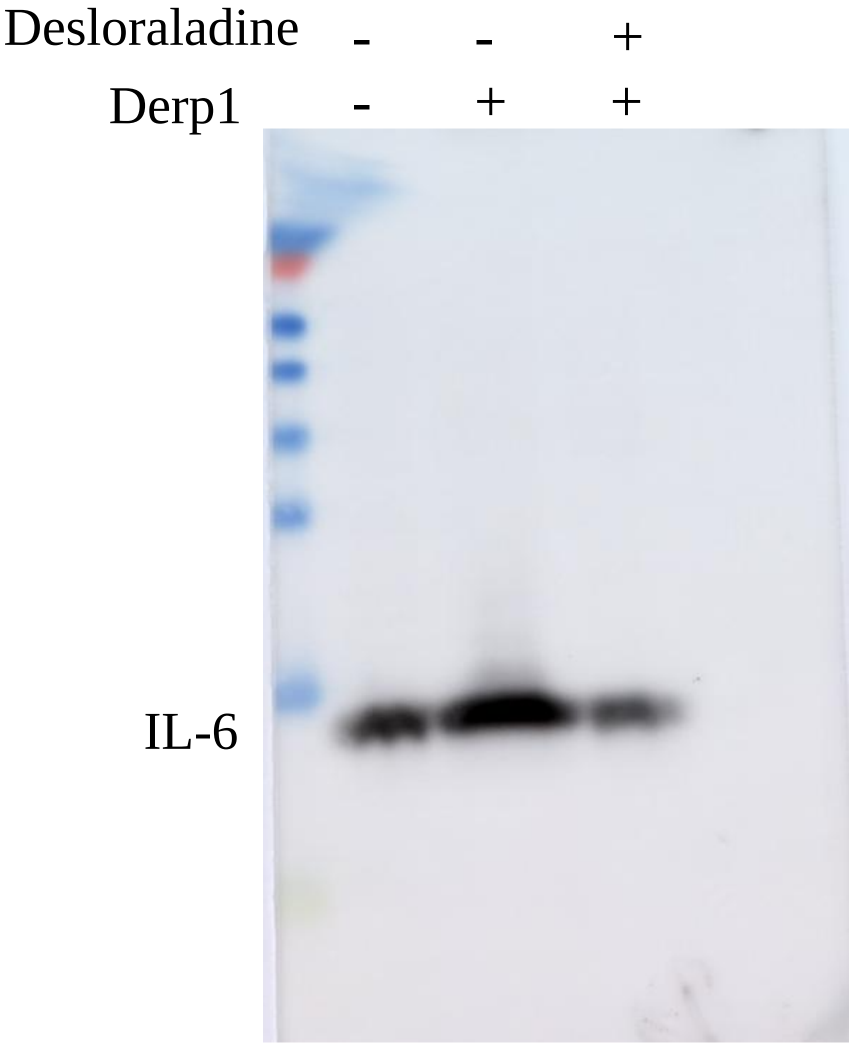
Desloraladine - - +
Derp1 - + +
IL-6

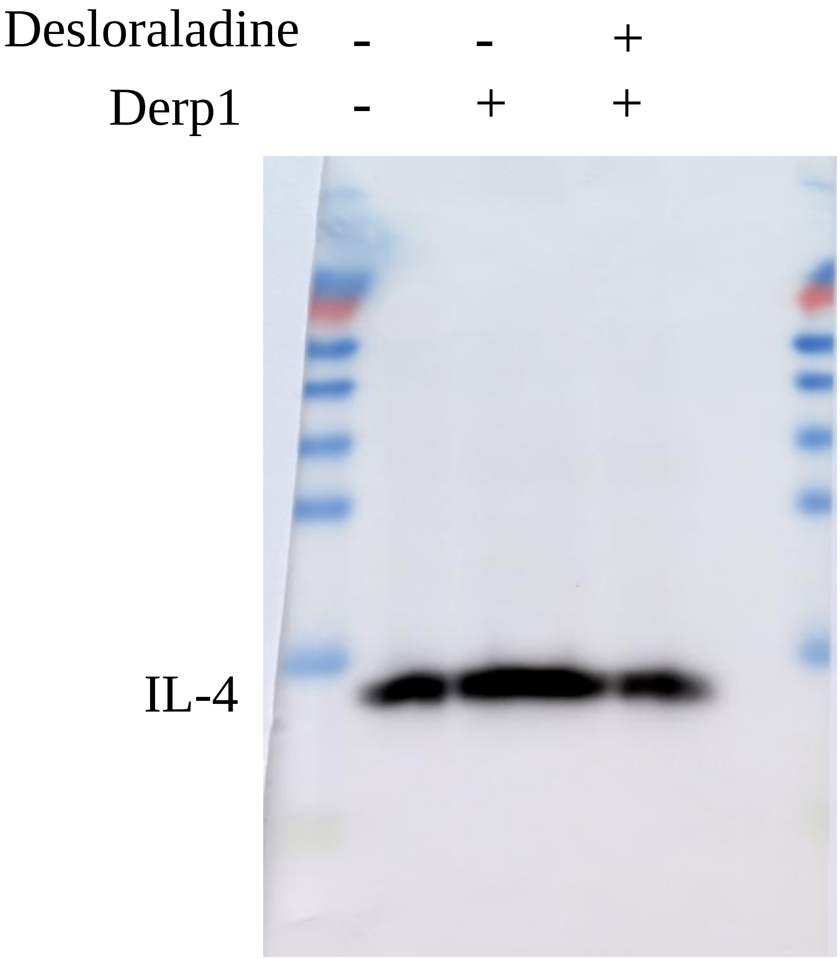
Desloraladine - - +
Derp1 - + +
IL-4

Desloraladine	-	-	+
Derp1	-	+	+

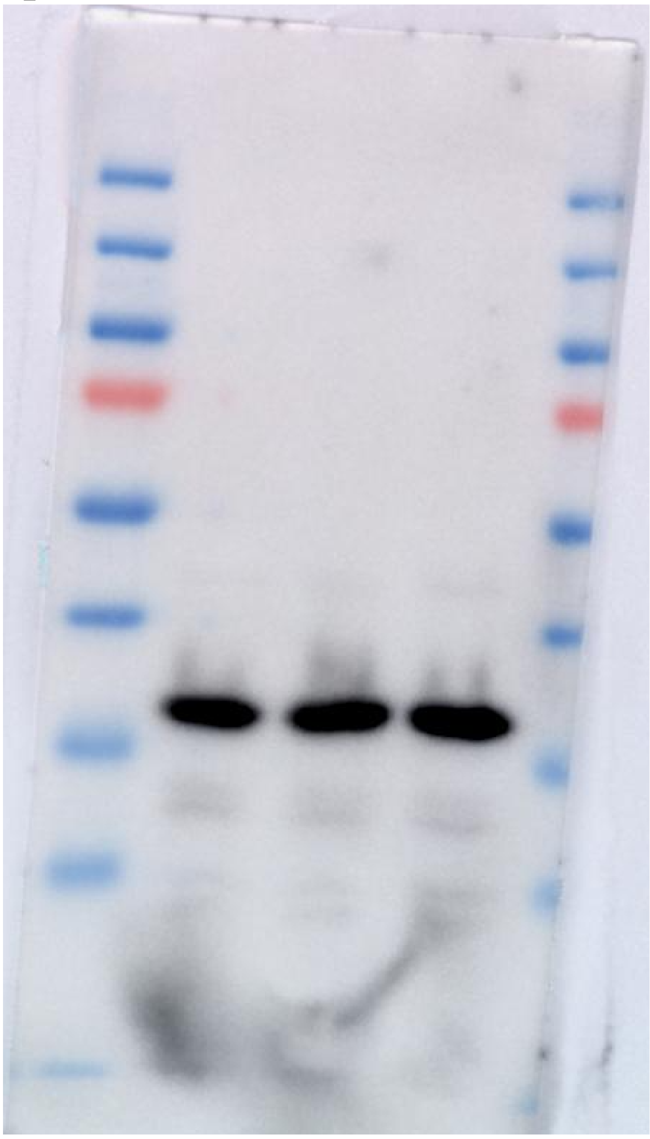

GAPDH
