# Supplementary material for: Study on the pathogenesis of PLXNB1 gene in olfactory dysfunction of allergic rhinitis
Source: PLoS One. 2026 Jun 1;21(6):e0350102. doi: 10.1371/journal.pone.0350102 (PMC13225367; doi:10.1371/journal.pone.0350102)
Supplement: Attachment — Submitted filename: Response to Reviewers.docx [file pone.0350102.s003.docx]

5. Review Comments to the Author

Please use the space provided to explain your answers to the questions above. You may also include additional comments for the author, including concerns about dual publication, research ethics, or publication ethics. (Please upload your review as an attachment if it exceeds 20,000 characters)

Reviewer #1: 1. Although the importance of the PLXNB1 gene in the nervous system has been partially revealed by previous studies, its specific role in olfactory dysfunction in patients with allergic rhinitis (AR) has not been fully investigated.

2. It is the first time to systematically explore the expression characteristics and potential mechanisms of the PLXNB1 gene in olfactory dysfunction caused by AR in this study.

3. During the development of AR, the nasal mucosa is stimulated by allergens, triggering an immune inflammatory response that damages olfactory neurons. The Plexin B1 protein encoded by the PLXNB1 gene plays a key role in neuronal development and repair. This gene may be regulated during inflammation, with downregulation or deficiency exacerbating olfactory dysfunction.

4. The interaction between the inflammatory response and changes in PLXNB1 function may create a vicious cycle, further worsening the reduction in odor perception, impairment of odor discrimination ability, and overall decline in olfactory quality. This mechanism suggests the potential value of PLXNB1 as a therapeutic target.

5. The p38 MAPK pathway, a key player in chronic inflammation and tissue damage, has been implicated in the release of inflammatory cytokines and airway remodeling in various respiratory diseases. In our study, siRNA-mediated PLXNB1 knockdown led to a significant increase in p-p38 levels, indicating that PLXNB1 may suppress inflammation by inhibiting this pathway. Importantly, we observed that Desloratadine not only reduced the expression of inflammatory cytokines but also upregulated PLXNB1 expression, implying that PLXNB1 may represent a previously unrecognized therapeutic target of traditional antihistamines. This finding expands our understanding of the pharmacological mechanisms of anti-allergic drugs and provides a theoretical basis for future targeted interventions in AR-associated olfactory dysfunction.

Response to Reviewer #1 We sincerely thank the reviewer for the careful evaluation of our manuscript and for the positive and constructive comments. We appreciate the reviewer’s recognition of the novelty and significance of our study. The reviewer’s comments mainly summarize the background, significance, and key findings of our work. As no specific revisions were requested, we have carefully checked the manuscript again and made minor language polishing to improve clarity. We are grateful for the reviewer’s insightful evaluation and support for our work. Thank you for your time and consideration.
